# Blunt Force Thoracic Trauma: A Case Study of Pericardial Rupture and Associated Cardiac Herniation

**DOI:** 10.1155/2014/946061

**Published:** 2014-08-12

**Authors:** O. S. Glotzer, A. Bhakta, T. Fabian

**Affiliations:** Section of Thoracic Surgery, Department of Surgery, Albany Medical Center, Albany, NY 12208-3403, USA

## Abstract

Pericardial rupture, with associated cardiac herniation, is generally fatal. Diagnosis is difficult and frequently missed due to the subtlety of identifying characteristics. We report a case of a left sided pericardial rupture and cardiac herniation resulting from a high speed motorcycle collision. This report describes the course of treatment from the emergent admission to the diagnosis of the pericardial tear to retrospective CT analysis and rupture identification. In addition the difficulties of initial diagnosis, key symptoms, and identification of CT images are presented and discussed.

## 1. Background

According to the Centers for Disease Control's most recent statistical analysis, traumatic injury and accidents are the 5th leading cause of death in the United States. Of these accidents, 25% include blunt force thoracic trauma [1]. Surprisingly, even with such a high incidence of blunt force thoracic trauma, only a few cases of pericardial rupture have been reported in the literature, the majority of which were discovered postmortem [2–5]. We present a case of blunt force thoracic trauma leading to a pericardial rupture, with acute cardiac herniation, which resulted in cardiogenic shock. The reported case not only demonstrates the difficulty of initial diagnosis but also highlights clinical and radiographic findings which could have identified the injury earlier in this patient's course.

## 2. Case Report

A 36-year-old male helmeted patient was transferred from an outside hospital following a high speed motorcycle accident in which he collided with another vehicle. Prior to transfer, the patient had a reported GCS of 14, was hemodynamically stable, and had suffered multiple rib and lower extremity fractures. Upon arrival to our trauma center, he became unresponsive and tachycardic and had a blood pressure of 56/42. He was intubated and underwent bilateral chest tube placement and aggressive fluid resuscitation. Focused Assessment with Sonography in Trauma (FAST) scan showed no evidence of intra-abdominal injury, but chest X-ray was suggestive of a possible left pleural effusion. CT images ruled out intracranial injury and spinal injury but did indicate bilateral hemopneumothorax. Following improvement in hemodynamic status, the patient underwent orthopedic surgery to address trauma to the lower extremities; intervention included intramedullary rodding of an open femur fracture and repair of a quadriceps tendon laceration. He tolerated the procedures well.

**Figure 1:**
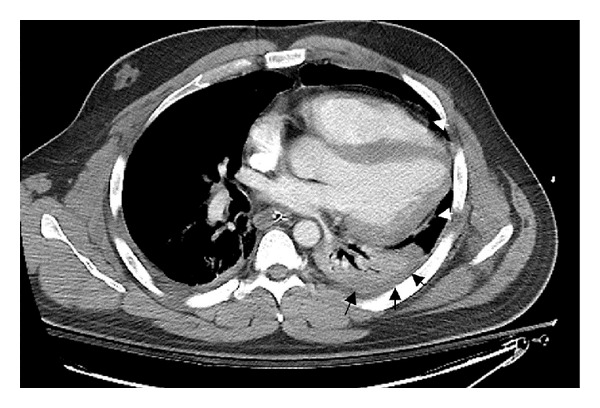
The CT study performed upon emergency department arrival. The heart and mediastinum are dramatically displaced to the left. The heart has migrated posteriorly to rest on the atelectatic left lower lobe of the lung (black arrows). The white arrows demarcate the edges of the pericardium through which the heart has herniated.

Although the patient remained hemodynamically stable he continued to be tachycardic, required mechanical ventilation, and remained in the surgical ICU. His postoperative course was complicated by episodes of severe tachypnea and tachycardia. On day 11 of the hospital course a rapid response was called for the second time in two days, and thoracic surgery was consulted for a persistent “left pleural effusion.” The patient had a heart rate of 160 with intermittent atrial flutter and systolic blood pressures of 100–110 mmHg. CT scan of the chest was consistent with an empyema, complete atelectasis of the left lower lobe, and a substantial shift of the mediastinum and heart into left chest (Figure 1). A left thoracotomy was performed. Dense fibrinopurulent debris was found throughout the left chest with entrapped upper and lower lobes. During decortication, a large vertical pericardial tear was identified running parallel to the phrenic nerve measuring 14 cm in length. This allowed for cardiac herniation through the defect, and the heart was found to be resting on the compressed lower lobe of the left lung.

Reduction of the hernia and repair of the defect were thought to be technically implausible and therefore the defect was left open. Total decortication was completed and the patient was left intubated for alveolar recruitment and full left lower lobe expansion. This maneuver allowed the heart to shift into a more native position.

**Figure 2:**
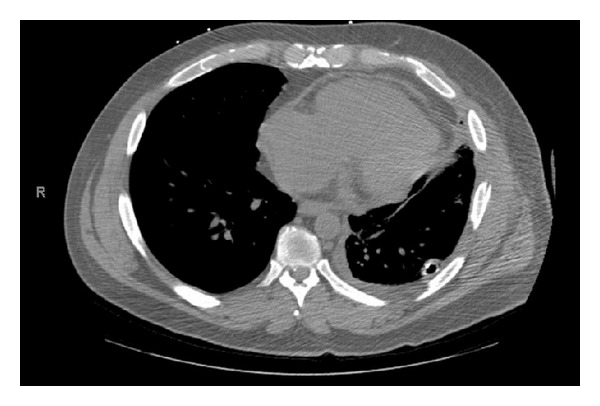
The CT study performed on postoperative day 7 that shows dramatic improvement in the cardiac position as well as decreased atelectasis of the lower lobe of the left lung.

The patient had significant improvement in all hemodynamic parameters following surgery with resolution of tachycardia and improved systolic pressures. The patient was extubated on postoperative day (POD) 4, and chest tubes were removed on PODs 4 and 5. Through the remainder of the hospital stay the patient showed progressive improvement (Figure 2) and was discharged home 25 days after admission, with a blood pressure of 106/60 and a heart rate of 67 beats per minute.

## 3. Discussion

Traumatic pericardial rupture is an uncommon finding with literature review putting the incidence at 0.3% out of all trauma cases; 68% are attributed to motor vehicle accidents and occur on the left side 64% of the time [6]. The reported survival rate for these injuries has been estimated to be as low as 24% and as high as 47% [1, 6]. Of the reported cases, 64% were fatal due to delayed diagnosis and the discovery of rupture and/or cardiac herniation was made at the time of autopsy [6].

Diagnosis of a pericardial rupture and the subsequent cardiac deviation is difficult in the trauma setting and is generally discovered during either exploratory surgery or other interventions for a related disorder. Symptoms and characteristics that are helpful in the diagnosis of cardiac herniation include a characteristic splashing murmur called a “Bruit de Moulin,” a hypotension that is unresponsive to fluid or pharmacological treatment and tachycardia [6]. The lethal aspect of cardiac herniation involves the torsion of the great vessels and subsequent loss of cardiac output.

**Figure 3:**
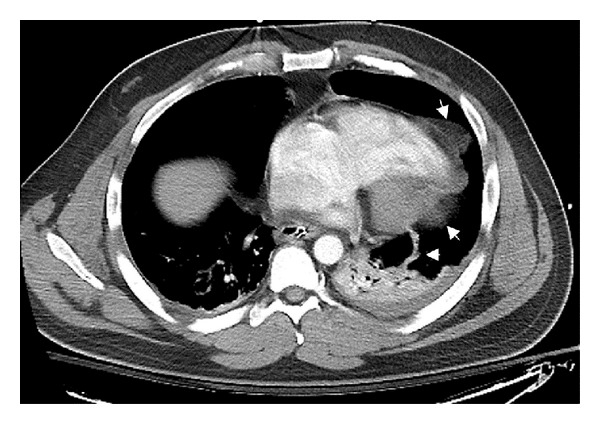
This CT study was performed shortly after arrival to the emergency room and shows the pericardium in an unnatural shape with the left ventricle herniating through the tear that runs vertically up the lateral aspect of the sac (white arrows).

In this case, the diagnosis was delayed for 11 days. It is the opinion of the authors that without intervention the patient would have continued to deteriorate, and this injury would have resulted in mortality. On review of the case some salient features come to light. It is clear that multiple trauma played a role in the delay of diagnosis. Tachycardia, relative hypotension, and hypoxemia are all extremely nonspecific and could result from a multitude of etiologies including pain, empyema, pulmonary emboli, and bone marrow emboli. Upon exclusion of these etiologies, other causes must be investigated. In hindsight the initial CT scan of the chest, which was performed within 1 hour of the injury, demonstrates a few critical features which support the radiographic diagnosis of pericardial rupture and cardiac herniation. First, the left lower lobe is completely collapsed in the absence of significant pleural fluid (Figure 1) which is unique immediately following injury. Second, the heart has dramatically shifted leftwards and rests on the left lower lobe. And finally there is an abnormal structure visualized inside the left hemithorax which later proved to be the ruptured pericardium (Figure 3). The mechanism of cardiac herniation was probably due to the development of atelectasis in the left lower lobe resulting from the initial pericardial injury. Most likely, the atelectatic lung allowed the heart to herniate through the pericardial defect into the newly created space in the left chest cavity. Even with the assistance of modern scans, it is extremely difficult to effectively diagnose a pericardial rupture preoperatively and the above observations were only made in retrospect.

## 4. Conclusion

Given the rarity of diagnosis and successful treatment of pericardial rupture and associated cardiac herniation, this case provides useful insight into a very unique condition. Due to its difficulty in recognition, pericardial rupture should be considered in all cases of blunt force chest trauma as it is likely lethal if gone untreated. Even with the aid of modern computerized tomography, successful preoperative diagnosis of a pericardial rupture with or without cardiac herniation is very difficult and has only seldom been reported in the literature [5, 7, 8]. Once confirmed, however, surgical intervention can be executed effectively as is described in the instance of this case report.
